# Ataxia Telangiectasia Diagnosed on Newborn Screening–Case Cohort of 5 Years' Experience

**DOI:** 10.3389/fimmu.2019.02940

**Published:** 2019-12-20

**Authors:** Amarilla B. Mandola, Brenda Reid, Raga Sirror, Rae Brager, Peter Dent, Pranesh Chakroborty, Dennis E. Bulman, Chaim M. Roifman

**Affiliations:** ^1^Division of Immunology and Allergy, Department of Paediatrics, The Hospital for Sick Children and the University of Toronto, Toronto, ON, Canada; ^2^The Canadian Centre for Primary Immunodeficiency and the Jeffrey Modell Research Laboratory for the Diagnosis of Primary Immunodeficiency, the Hospital for Sick Children, Toronto, ON, Canada; ^3^Paediatric Allergy/Immunology, Thunder Bay Regional Health Sciences Center, North Ontario School of Medicine, Thunder Bay, ON, Canada; ^4^Division of Rheumatology, Immunology, and Allergy, Department of Paediatrics, McMaster Children's Hospital, McMaster University, Hamilton, ON, Canada; ^5^Department of Pediatrics, CHEO Research Institute and Newborn Screening Ontario, University of Ottawa, Ottawa, ON, Canada

**Keywords:** ataxia telangiectasia, newborn screen, primary immunodeficiency, TRECs, lymphopenia

## Abstract

Ataxia telangiectasia (AT) is a genetic condition caused by mutations involving *ATM* (Ataxia Telangiectasia Mutated). This gene is responsible for the expression of a DNA double stranded break repair kinase, the ATM protein kinase. The syndrome encompasses combined immunodeficiency and various degrees of neurological abnormalities and increased risk of malignancy. Typically, patients present early in life with delay in neurological milestones, but very infrequently, with life threatening infections typical of a profound T cell deficiency. It would therefore be unexpected to identify this condition immediately after birth using T cell receptor excision circle (TREC)-based newborn screening (NBS) for SCID. We sought to evaluate the frequency of AT detected by NBS, and to assess immunity as well as the genetic aberrations associated with this early presentation. Here, we describe the clinical, laboratory, and genetic features of patients diagnosed with AT through the Ontario NBS program for SCID, and followed in our center since its inception in 2013. Four patients were diagnosed with AT as a result of low TRECs on NBS. In each case, whole exome sequencing was diagnostic. All of our patients had compound heterozygous mutations involving the FRAP-ATM-TRRAP (FAT) domain of the *ATM* gene, which appears critical for kinase activity and is highly sensitive to mutagenesis. Our patients presented with profound lymphopenia involving both B and T cells. The ratio of naïve/memory CD45+RA/RO T cells population was variable. T cell repertoire showed decreased T cell diversity. Two out of four patients had decreased specific antibody response to vaccination and hypogammaglobulinemia requiring IVIG replacement. In two patients, profound decreased responses to phytohemagglutinin stimulation was observed. In the other two patients, the initial robust response declined with time. In summary, the rate of detection of AT through NBS had been surprisingly high at our center. One case was identified per year, while the total rate for SCID has been five new cases per year. This early detection may allow for better prospective evaluation of AT shortly after birth, and may assist in formulating early and more effective interventions both for the neurological as well as the immune abnormalities in this syndrome.

## Introduction

The protein kinase Ataxia Telangiectasia Mutated (ATM) is a high molecular weight PI3K-family kinase involved in the phosphorylation of multiple proteins, including in key cellular functions such as gene transcription and expression, response to oxidative stress, and energy metabolism. Together, ATM acts as a temporal gate keeper for proper cell division and appropriate repair (1–3).

Ataxia telangiectasia (AT) is an autosomal recessive syndrome encompassing progressive neuronal degeneration, ocular and cutaneous telangiectases, variable immunodeficiency, and cancer susceptibility. The ESID diagnostic criteria for AT includes ataxia and at least two of the following features: (a) oculo-cutaneous telangiectasia, (b) elevated alpha-fetoprotein (AFP), (c) typical AT karyotype (translocation of chromosomes 7; 14), (d) cerebellum hypoplasia on MRI (4). The criteria do not include neurological abnormalities, likely because of its inconsistency, great variability, and due to challenges in obtaining data from pediatric examination.

By controlling cell cycle and DNA repair, ATM plays an important role in the development and function of both the cellular and humoral immune system. The development of appropriate T cell receptor (TCR) repertoire is dependent on the repair of V(D)J recombination-induced breaks by the non-homologous end-joining (NHEJ) pathway, which is promoted by ATM (5, 6). Proper class-switch recombination is facilitated by ATM, through the correction of breaks by a Ku-dependent end-joining pathway, as well as preventing aberrant translocations due to double strand breaks and propagating the Ku-independent alternative NHEJ (A-NHEJ) pathway (5, 7, 8).

Diagnosis of AT patients may be delayed due to the wide variability in clinical phenotype; the syndrome is frequently confused with cerebral palsy and the immunological evaluation overlooked or misdiagnosed as Hyper-immunoglobulin M syndrome (9–12).

Review of the literature reveals that the immunological presentation of AT is highly variable; IgA concentrations are low in more than 50% of cases, IgM levels are elevated in up to 60% of cases, but low IgG is infrequent (occurring in 10–18% of cases). Numbers of T cells, in particular CD4+ cells, may decline over time in 30–75% of cases. However, profound lymphopenia in neonates was not well-recognized prior to the introduction of NBS (13–15), and the AT cases that have since been identified by TREC/KREC in infants (16–18) are likely an underestimate of the true number of patients affected. The complete absence of ATM enzyme activity is much more likely to result in clinical and/or immunological features of immunodeficiency compared to those who retain residual activity (19).

We describe here four patients with AT identified by the NBS system in Ontario, Canada.

## Methods

### Patients

This study conformed to the Declaration of Helsinki and all local ethical requirements. Information on presentation, complications, laboratory parameters, management, and outcomes were compiled both prospectively and retrospectively using parent interview and medical chart review. All laboratory results were analyzed with reference to age-related normal ranges. Written informed consent was obtained from the parents or guardians of the participant for the publication of this study.

### TREC Determination From Guthrie Blood Spots

Methodology for the assessment of TREC levels by qPCR was performed as previously described (20).

### Lymphocyte Proliferation

Lymphocyte proliferative responses were assayed to mitogens including phytohemagglutinin (PHA) and anti-CD3. All assays were performed in triplicate and were compared with simultaneously stimulated normal controls, as previously described (21).

### Chromosome Breakage Analysis and G-Band Evaluation

For chromosome spontaneous breakage frequency evaluation, fifty metaphases were examined by solid stain analysis. One hundred metaphase cells from a 3-day PHA-stimulated lymphocyte culture were examined by G-band analysis. Chromosome rearrangements involving regions 7p14, 7q34, and 14q11.2 were evaluated in metaphase cells.

### Whole Exome Sequencing and Variant Calling

DNA from blood was submitted to The Center for Applied Genomics (TCAG), Toronto, Canada for exome library preparation and sequencing. DNA was quantified by Qubit DNA HS assay (Life Technologies, Carlsbad, CA) and 100 ng of input DNA was used for library preparation using the Ion AmpliSeq Exome Kit (Life Technologies) according to the manufacturer's recommendations. The Ampliseq Exome library was immobilized on Ion PI™ Ion Sphere™ particles using the Ion PI Template OT2 200 Kit v3. Sequencing was performed with the Ion PI Sequencing 200 Kit v3 and Ion PI Chip v2 in the Ion Proton™ semiconductor sequencing system following the manufacturer's recommendation. Alignment and variant calling were performed using Torrent Suite (v4.0) on the Ion Proton Server, using the Ion Proton AmpliSeq germline low stringency setting and the hg19 reference genome. The variants were annotated using an in-house annotation pipeline (22) based on Annovar (November 2014 version) (23) and RefSeq gene models (downloaded from UCSC 01 August 2015).

### Sanger Sequencing

Patients' genomic DNA was extracted from peripheral blood lymphocytes using the Geneaid Genomic DNA Mini Kit. Genomic DNA was amplified by PCR with specific primers designed upstream and downstream of the ATM gene. Sequencing was done using GenomeLab Dye Terminator Cycle Sequencing (DTCS) Quick Start Kit (Beckman Coulter) and analyzed on CEQ 8000 Genetic Analysis System (Beckman Coulter).

## Results

**Figure 1:**
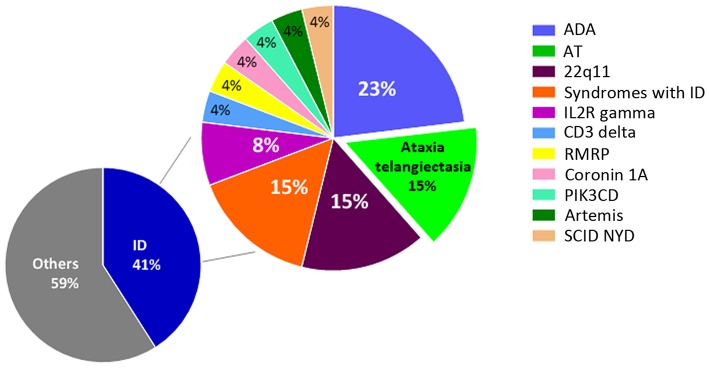
Positive NBS results for SCID at the Hospital for Sick Children from 2013 to 2018. A total of 63 infants were NBS positive for SCID at our center. Of the 26 cases with confirmed immunodeficiency, 23% were diagnosed with adenosine deaminase deficiency (ADA), 15% with ataxia telangiectasia (AT), 15% with syndromes involving immunodeficiency (ID), 8% with IL2R gamma deficiency, and 4% each with deficiencies of CD3 delta, RMRP, coronin 1A, PIK3CD, Artemis, and SCID not yet defined (NYD). The remaining 37 cases were associated with maternal immunosuppression, cardiac post-thymectomy, prematurity, and lymphopenia NYD.

NBS for Severe Combined Immunodeficiency (SCID) was first introduced in Ontario, Canada, in 2013, and has since been expanded to the Maritime provinces of New Brunswick, Nova Scotia, and Prince Edward Island in 2016. This lifesaving test has yet to be introduced in other jurisdictions of Canada. In Ontario, where there are ~140,000 births/year, an unexpectedly high number of patients with AT were diagnosed at our center. The Hospital for Sick Children, Toronto, Ontario, is a quaternary hospital with a catchment area of 10 million people. Over the past 5 years, 63 infants at our center were screened positive for SCID on NBS for various reasons (Figure 1). At this point we can conclude that primary immunodeficiencies are more common than estimated in the past, and recent reports suggest that around 1% of the global population may be affected (4, 24–26). The prevalence of AT is estimated to be between 1 in 40,000 and 1 in 100,000 live births, though in certain populations the frequency of mutations are different due to founder effects (14, 27).

### Patients

#### Patient 1

**Figure 2:**
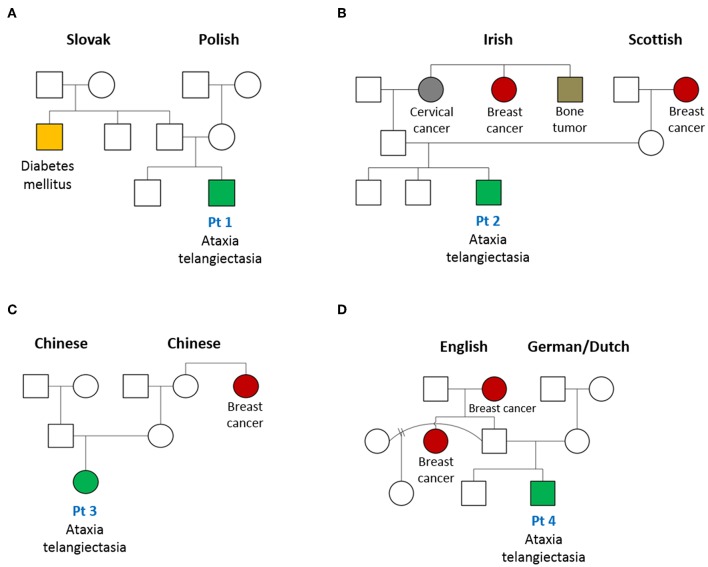
Pedigree of patients with AT diagnosed on newborn screen for SCID. Family tree of patients (Pt) 1–4 (**A–D**, respectively) in this case series are shown. An increased frequency of cancer, especially breast cancer, in female family members is noted.

Patient 1 is the second-born child of a non-consanguineous union with parents of Eastern European origins, with unremarkable family history except for diabetes in a paternal uncle (Figure 2A). His first physical examination at 3 weeks of age was unremarkable, with normal head circumference (30th percentile). At age 12 months, he developed a wobbly gait with mild hypotonia and at 18 months he had oculomotor apraxia, excessive drooling indicative of oro-motor apraxia, and appendicular hypotonia. He had slower baseline gait than expected for age, and truncal ataxia was prominent when walking. Delayed motor development was also observed. His brain MRI was normal. Lymphocyte immunophenotyping revealed reduced numbers of CD19+ B cells, CD3+, CD4+, and CD8+ T cells, which further declined at 8 months of age (Table 1). The number of CD4+ naïve T cells were diminished, suggesting ineffective thymic production or egress of these cells (Table 2). His T cell repertoire showed decreased diversity, while lymphocyte proliferation responses to PHA were preserved. His humoral evaluation at that time showed normal age referenced immunoglobulin levels, however, the patient's family decided against vaccination, and thus specific antibody titers were not assessed. His AFP level was slightly above normal.

**Table 1:** Initial immune evaluation at SCID retrieval.

	**Pt 1**	**Pt 2**	**Pt 3**	**Pt 4**	**Reference range**
White blood cell count	6.4	3.05	5.4	5.7	5–20 × 10^9^cells/μL
Neutrophil count	2.6	0.55	2.21	2.91	1–9.5 × 10^9^cells/μL
Lymphocyte count	**1.8**	**1.52**	**1.73**	**1.22**	2–17 × 10^9^cells/μL
Eosinophil count	0.8	0.25	0.22	0.83	0.07–1 × 10^9^cells/μL
Thrombocyte count	440	323	547	**737**	150–400 × 10^9^cells/μL
CD3+	**888**	**780**	**1142**	**664**	2,300–6,500 cells/μL
CD19+	**234**	**79**	**43**	**154**	600–3,000 cells/μL
CD3+/CD4+	**633**	**262**	**349**	**421**	1,500–5,000 cells/μL
CD3+/CD8+	**233**	**485**	759	**228**	500–1,600 cells/μL
NK	528	531	440	258	100–1,300 cells/μL
PHA (Stimulation Index, SI)	563	**416**	**151**	**158**	>450 SI
CD3 Mitogen Stimulation	ND	Normal	**7.2 (C:216)**	**9.3 (C:255)**	

*C, control; ND, not determined; bold text indicates values that fall outside of the reference range*.

**Table 2:** Immunological evaluation at age 8 months.

	**Pt 1**	**Pt 2**	**Pt 3**	**Pt 4**	**Reference range**
White blood cell count	6.18	5.13	3.2	3.4	5–20 × 10^9^cells/μL
Neutrophil count	2.8	2.9	1.09	0.8	1–9.5 × 10^9^cells/μL
Lymphocyte count	**1.7**	**1.27**	**0.34**	**1.5**	2–17 × 10^9^cells/μL
Eosinophil count	0.38	0.1	0.26	0.4	0.07–1 × 10^9^cells/μL
Thrombocyte count	510	394	466	614	150–400 × 10^9^cells/μL
CD3+	**645**	**677**	**664**	**823**	2,300–6,500 cells/μL
CD19+	**118**	**186**	**116**	**201**	600–3,000 cells/μL
CD3+/CD4+	**466**	**370**	**441**	**550**	1,500–5,000 cells/μL
CD3+/CD8+	**109**	**242**	**182**	**235**	500–1,600 cells/μL
NK	685	531	581	547	100–1,300 cells/μL
CD4+/CD45+ RA+	**10 (C:44)**	**16.4 (C:34)**	**11 (C:33)**	**14 (C:23)**	
CD4+/CD45+ RO+	38 (C:17)	39.8 (C:19)	10 (C:12)	20 (C:9)	
IgG	2.8	5.5	**<1.1**	**<1.1**	1.1–7.0 g/L
IgM	0.7	0.2	**1**	**1.2**	0.1–0.7 g/L
IgA	0.1	0.1	**<0.1**	**<0.1**	0.1–3 g/L
Anti-pneumococcal Ab	Not vaccinated	11	on IVIG	on IVIG	
Anti-tetanus toxoid Ab	Not vaccinated	2.86	0.16	0.03 (5 mo)	>0.1
Diphtheria toxoid Ab	Not vaccinated	3	on IVIG	on IVIG	>0.01
AFP	**23**	**56**	**152**	**70**	<21 ng/mL

*Ab, antibody; C, control; IVIG, intravenous immunoglobulin; mo, months; bold text indicates values that fall outside of the reference range*.

#### Patient 2

Patient 2 is the third child born to a non-consanguineous family of English extraction, with a family history of breast cancer in the maternal grandmother and paternal aunt. In addition, the paternal grandmother was diagnosed with cervical cancer and a paternal uncle with a bone tumor (Figure 2B). His first physical examination at age 4 weeks was remarkable for mild axial hypotonia which continued to progress to wobbly gait, stagger, ataxia and mild diffuse hypotonia which were detected at 10 months. The patient's initial immune evaluation as a newborn revealed lymphopenia including reduced numbers of CD19+ B cells, CD3+, CD4+, and CD8+ T cells (Table 1). A repeat evaluation at age 8 months showed progressive lymphopenia with a reduction in CD4+ naïve T cells as well as reduced T cell repertoire diversity (Table 2). Lymphocyte responses to PHA were decreased. His immunoglobulin levels at both 8 and 18 months of age were normal, with sustained specific antibody titres to diphtheria and tetanus.

#### Patient 3

Patient 3 is the first-born child of a non-consanguineous family of Chinese ethnicity, with a family history of breast cancer in the maternal aunt (Figure 2C). Her first physical examination at age 3 weeks revealed axial hypotonia and her head circumference was below the 5th percentile for age, where it remained thereafter. At 12 months of age, profound hypotonia, nystagmus, unbalanced gait, and feeding problems became apparent, and at 18 months she was found to have additional neurological features including dysarthria, dysmetria, nystagmus, ataxia, and oculo-oro-motor apraxia. The patient's initial immune evaluation while a newborn demonstrated reduced numbers of CD19+ B cells, CD3+, CD4+, and CD8+ T cells which further declined when assessed at 8 months of age (Table 1). The number of CD4+ naïve T cells were reduced and she had a limited T cell repertoire (Table 2). Her lymphocyte proliferation responses to PHA were remarkably depressed. Humoral evaluation at age 8 months showed severe hypogammaglobulinemia including low IgG (IgG < 1.1), low IgA level (IgA < 0.1), elevated IgM (IgM = 1), and undetectable antibody responses to vaccines, requiring immunoglobulin replacement. Her AFP at age 8 months was greatly elevated (152).

#### Patient 4

Patient 4 is the second-born child of a non-consanguineous family of European origin, with a family history of breast cancer in a paternal aunt and grandmother (Figure 2D). His initial physical examination at age 3 weeks was unremarkable, but at 12 months he developed a wobbly gait with mild hypotonia, and at 18 months he had ataxia with unstable stance and gait, drooling, and oro-motor apraxia. The patient's initial immune evaluation while a newborn revealed reduced numbers of CD19+ B cells, CD3+, CD4+, and CD8+ T cells (Table 1), with further decline observed at 8 months (Table 2). His T cell repertoire was limited, and lymphocyte proliferation responses to PHA were very low. He had severe hypogammaglobulinemia including low IgG (IgG < 1.1) and low IgA level (<0.1), elevated IgM (1.2), and undetectable titers to childhood vaccines, requiring immunoglobulin replacement. His AFP at age 8 months was very elevated (70 ng/mL).

### Genetic Work Up

Following the finding of a positive NBS for SCID, and in the context of broader presenting features, we performed G-band analysis that, in each of these patients, demonstrated significant chromosome re-arrangements involving T cell receptor gamma, beta, and alpha/delta gene loci at chromosome locations 7p14, 7q35, and 14q11.2, and elevated frequency of spontaneous breakage. Whole exome sequencing (WES) revealed compound heterozygous mutations in the *ATM* gene in each patient (Table 3). The mutations were confirmed by Sanger sequencing and segregation studies showed that parents were heterozygous carriers of those mutations.

**Table 3:** SCID NBS TREC levels and genetic evaluation results.

	**Pt 1**	**Pt 2**	**Pt 3**	**Pt 4**
TRECs (copies/ 3 μL DNA) (cut-off >75 copies/3 μL)	**22**	**23**	**26**	**41**
WES/Sanger sequencing	c.331+1G>A; c.6095G>A	c.170G>A c.6997dupA	c.6679C>T c.7090-1G>A	c.5228C>T c.6908dupA
Affected region	FAT domain HEAT repeats	FAT domain HEAT repeats	FAT domain FAT domain	FAT domain FAT domain
G-band analysis assay	**Positive**	**Positive**	**Positive**	**Positive**

Bold text indicates values that fall outside of the reference range.

In Patient 1, WES revealed a c.331+1G>A mutation predicting p.Ser111Asn amino acid change affecting a splice donor site, and possibly disrupting the HEAT (Huntingin, elongation factor 3, protein phosphatase 2A, TOR1) domain. The second mutation, c.6095G>A, predicting p.Arg2032Lys amino acid change involves the FAT (Focal adhesion kinase targeting) domain. In Patient 2, two pathogenic variants, c.170G>A (p.Trp57^*^) and c.6997dupA (p.Thr2333Asnfs^*^40), involving both the HEAT and FAT domains were identified. Genetic evaluation of Patient 3 revealed two mutations within the FAT domain, c.6679C>T, (p.Arg2227Cys; pathogenic), c.7090-1G>A (p.Lys2363Arg; novel). Similarly, in Patient 4, the mutations c.5228C>T (p.Thr1743Ile; likely pathogenic) and c.6908dupA (p.Glu2304Glyfs^*^69; pathogenic) were both localized to the FAT domain.

## Discussion

The implementation of TREC-based SCID NBS in Ontario, Canada, has enabled the early detection and diagnosis of SCID that would otherwise be missed or delayed until the onset of life-threatening infections. Unfortunately, it appears that many cases of significant T cell deficiencies cannot be detected by this methodology. Surprisingly, some non-SCID conditions have been rarely detected by NBS (28). AT has not been typically regarded as having a SCID-like clinical course or fate.

In our cohort, we describe four patients with AT who all presented with low TRECs on SCID NBS. The initial approach to patients with an abnormal SCID NBS in Canada is described in Biggs et al. (29). All had profound, sustained B and T cell lymphopenia, which is consistent with low thymic output. Our patients had low naïve CD4+/CD45+ RA+ populations compared to age appropriate controls. Three patients presented with decreased lymphocyte proliferation responses. Two out of the four patients showed early onset humoral immunodeficiency and were started on immunoglobulin replacement therapy.

Patients with AT are rarely diagnosed in the first year of life, largely because their typical neurological manifestations are noted at a later age. Many are incorrectly diagnosed with cerebral palsy. Early detection at the newborn age leads to the correct diagnosis and might aid in early interventions. However, this may pose an ethical conundrum since some jurisdictions, such as the Netherlands, do not allow the screening and reporting of diseases for which there is no cure. In Ontario, the finding of a positive SCID newborn screen, regardless of underlying cause, triggers urgent follow-up evaluation in accordance with our Ministry of Health-approved algorithm for assessment and treatment of such cases (30).

Each of our patients carry compound heterozygous mutations in the *ATM* gene, one of which is a pathogenic variant localized to the FAT domain. As expected, patients' pedigrees also show an increased frequency of cancer, especially breast cancer in female family members.

The detection of AT by NBS was first reported in 2012 in Swedish newborns (6) and subsequently in other jurisdictions where NBS for SCID has been implemented (31, 32). We have shown here that AT, if detected early by TREC-based NBS, has a more profound immunological and neurological phenotype, and this intuitively might predict a more severe disease course.

Early genetic diagnosis of AT enables very early patient centered and individualized interventions, including physiotherapy, neurological support, proper immunological evaluation, and infection prevention before onset of complications (PJP prophylaxis and IVIG before the development of bronchiectasis). Moreover, it may also aid in further understanding possible genotype-phenotype correlations.

## Conclusion

We have demonstrated that AT can be detected shortly after birth by NBS for SCID. AT should be considered in cases with NBS positivity because of its relatively high frequency, as shown in our cohort (15%). This early detection allows for early referral to specialized centers for comprehensive evaluation and guidance. It enables the provision of individualized intervention for patients as well as genetic counseling for the family members, especially mothers, who, as carriers, have an increased risk of developing breast cancer and other malignancies. Furthermore, the early detection of immune abnormalities allows for appropriate treatment to prevent or minimize the otherwise complicated disease course that AT patients may suffer.

## Data Availability Statement

The raw data supporting the conclusions of this study will be made available upon request.

## Ethics Statement

The studies involving human participants were reviewed and approved by the Research Ethics Board, Hospital for Sick Children. Written informed consent to participate in this study was provided by the participants' legal guardian/next of kin.

## Author Contributions

CR and AM conceptualized and designed the study and wrote the initial draft of the manuscript. BR, RS, RB, and PD were involved in the clinical care of the patients. DB and PC evaluated NBS TRECs. All authors contributed to manuscript revision, read, and approved the submitted manuscript.

### Conflict of Interest

The authors declare that the research was conducted in the absence of any commercial or financial relationships that could be construed as a potential conflict of interest.
